# Red Cell Distribution Width in Chronic Liver Disease: An Observational Study

**DOI:** 10.7759/cureus.40158

**Published:** 2023-06-08

**Authors:** Sankar Kalairajan, Kavitha K. K., Govindaraj P.

**Affiliations:** 1 Department of Internal Medicine, Aarupadai Veedu Medical College and Hospital, Vinayaka Mission's Research Foundation (DU), Pondicherry, IND; 2 Department of Microbiology, Swamy Vivekanandha Medical College Hospital and Research Institute, Tiruchengode, IND

**Keywords:** child-pugh score, meld score, red cell distribution width, chronic liver disease, ascites

## Abstract

Background: Chronic liver diseases (CLDs) encompass a group of conditions that are marked by diminished liver function due to ongoing inflammation or damage. This study aimed to establish a relationship between the red cell distribution width (RDW) and two scoring systems, namely the Model for End-Stage Liver Disease (MELD) score and Child-Turcotte-Pugh (CTP) score, in individuals diagnosed with CLDs.

Methods: The study was carried out at Aarupadai Veedu Medical College & Hospital, Pondicherry, India, following approval from the Institutional Ethical Committee in the Department of General Medicine and Gastroenterology. It involved 50 patients aged 18 years and above who were diagnosed with CLD. The RDW of all selected patients was measured using a three-part autoanalyzer, and its correlation with the MELD and CTP scores was examined. Data analysis was performed using IBM SPSS (Statistical Package for Social Sciences), version 21.0 (IBM Corp., Armonk, NY), with a significance level set at p < 0.05.

Results: When comparing the baseline characteristics including age, gender, and encephalopathy, no statistically significant differences were found between RDW-standard deviation (RDW-SD) and RDW-corpuscular value (RDW-CV) (p > 0.05). However, a statistically significant correlation was observed between the presence of ascites and RDW-CV values (p = 0.029). Furthermore, there was a significant association between the CTP score and RDW-SD (p < 0.0001). The association between the MELD score and RDW-SD was also found to be statistically significant (p = 0.006). Similarly, statistically significant results were obtained between the MELD score and RDW-CV (p = 0.034).

Conclusion: The utilization of RDW holds promise as a convenient and effective tool for evaluating the severity of individuals with CLD.

## Introduction

Chronic liver disease (CLD) has become a significant global health concern, associated with a rise in morbidity and mortality rates. In 2013 alone, CLD accounted for 1.75 million recorded deaths worldwide [1]. CLD arises from various underlying causes, impairing both the biochemical and physiological functions of the liver [1-6]. While acute liver damage is often reversible, chronic liver injury can lead to persistent issues such as fibrosis, hepatic inflammation, portal hypertension, cirrhosis, and hepatocellular carcinoma (HCC). During the initial stages of the disease, hepatocytes undergo a reparative process, but continuous tissue damage eventually leads to fibrosis and the accumulation of extracellular matrix (ECM) in the perisinusoidal space of Disse [7,8].

Hepatic fibrosis can be a common pathway among various etiologies, including viral hepatitis B and C infections, nonalcoholic steatohepatitis (NASH), and alcohol-related liver disease [9]. If left untreated, fibrosis can progress to cirrhosis and ultimately HCC. Among the various cellular components involved, stellate cells play a crucial role in the repair and regeneration of damaged hepatocytes. These cells are responsible for regulating processes such as the synthesis and degradation of ECM, retinoid metabolism, vasoregulation, cytokine secretion, immune system regulation, lipid metabolism, and detoxification. In the context of chronic liver injury, stellate cells undergo phenotypic activation [10].

Portal hypertension is a frequently encountered complication in CLD. Understanding the presence and severity of portal hypertension is crucial for assessing disease progression and prognosis. Calculating hepatic vein pressure serves as an effective diagnostic method to evaluate and classify the severity of liver disease.

Red cell distribution width (RDW) is a parameter that quantifies the variation in size among circulating erythrocytes. Numerous studies have demonstrated a correlation between higher RDW values and an increased risk of mortality [11,12]. Within the liver, ferritin plays a role in regulating the expression of pro-inflammatory cytokines. In the context of CLD, elevated levels of cytokines contribute to increased heterogeneity in the maturation of red blood cells (RBCs), resulting in elevated RDW values [13]. Inflammation and elevated serum ferritin are often linked to CLDs, which can result in the development of fibrosis and cirrhosis [4,5]. The activation of profibrogenic hepatic stellate cells (HSCs) and the elevation of ferritin levels are directly influenced by inflammatory mediators like TNFα and IL-1α. Earlier research has indicated the presence of a highly specific receptor for ferritin on activated HSCs, highlighting its strong affinity. Moreover, these studies have revealed that the binding of ferritin to HSCs relies on H-ferritin [7]. The involvement of ferritin in the regulation of HSC activation and the subsequent fibrogenesis processes has not been explored in previous investigations. Hepatic fibrosis is characterized by the transition of quiescent HSCs into a myofibroblastic phenotype, which manifests proliferative and proinflammatory traits. In response to chemokines and cytokines, HSCs exhibit migratory behavior toward the site of hepatic injury. At the injury site, HSCs play a direct role in depositing extracellular scar matrices, including collagens I, III, and IV; fibronectin; elastin; and laminin.

The Child-Turcotte-Pugh (CTP) score is a measurement utilized to assess the degree of liver injury and determine the prognosis of individuals affected by CLD [4]. This scoring system employs five variables to categorize patients into mild, moderate, and severe stages. On the other hand, the Model for End-Stage Liver Disease (MELD) score is employed to assess the outcomes of trans-jugular intrahepatic portosystemic shunt (TIPS) procedures [4,5]. The MELD score relies on three factors, and they are serum bilirubin, the international normalized ratio (INR), and serum creatinine levels [5]. The objective of the present study was to identify a straightforward, easily accessible, and cost-effective method for evaluating the severity of CLD. Thus, the study aims to establish a correlation between RDW and both the MELD and CTP scores in patients diagnosed with CLDs.

## Materials and methods

The cross-sectional study was conducted at Aarupadai Veedu Medical College & Hospital, Pondicherry, India, from July 2019 to June 2020 following approval from the Institutional Ethical Committee under letter number “AVMC/18/03” in the Department of General Medicine and Gastroenterology. The study enrolled a total of 50 patients diagnosed with CLD who were above 18 years of age. Patients with CLD may present with jaundice, confusion, abdominal distension, or easy bruising. The key findings on physical examination of a patient with CLD include sarcopenia, spider angiomata, a firm liver edge, splenomegaly, palmar erythema, and parotid enlargement. Prior to participation, written consent was obtained from all patients, and they were duly informed about the study’s purpose and procedures. Exclusion criteria comprised patients below 18 years of age, individuals with anemia attributed to other hematological causes (both microcytic and macrocytic), and those with known chronic renal disease, hypothyroid disorder, stroke, cardiac disease, peripheral artery disease, or pulmonary hypertension.

Blood samples were collected from all patients diagnosed with CLD within 24 hours of their admission. After one month, a follow-up was conducted via telephone. The severity of liver disease at admission was assessed using the MELD score. The MELD score utilizes the patient’s serum bilirubin and creatinine levels, as well as the INR for prothrombin time, to predict survival. On the other hand, the CTP score is employed to assess the prognosis of CLD, particularly cirrhosis. This score incorporates five clinical measures of liver disease, namely total bilirubin, serum albumin, prothrombin time, ascites, and hepatic encephalopathy.

In patients with CLD, RDW measurements were obtained using a three-part autoanalyzer. Additionally, various investigations were conducted, including liver function tests, renal function tests, prothrombin tests, INR, and serum albumin. The MELD score and CTP score were calculated. Data were tabulated on Microsoft Excel, and analysis was performed utilizing IBM SPSS (Statistical Package for Social Sciences), version 21.0 (IBM Corp., Armonk, NY). The parametric data were analyzed using the Student’s t-test, and the nonparametric data were analyzed using the chi-square test. A p-value of <0.05 was considered as statistically significant.

## Results

Among the individuals with CLD, the largest proportion (38%) fell within the 41-50 age-group. The male-to-female ratio was 86:14. Analysis of the data revealed that seven patients (14%) had an RDW-standard deviation (RDW-SD) below 46 femtoliters (fL). In the RDW-SD ranges of 50.1-60 and 60.1-70 fL, 13 patients (26%) were observed in each group. Regarding RDW-corpuscular volume (RDW-CV), seven patients exhibited values below 14.6%, while 12 patients had values exceeding 20.6%. In terms of ascites severity, 78% of patients had a moderate grade, while 8% had severe ascites. The most frequent CTP score range was between 10 and 15, observed in 28 patients (56%). Among the patients, 36% had no encephalopathy (grade 0), and a MELD score between 10 and 19 was observed in 22 patients (44%) (Table 1). 

**Table 1:** Patient characteristics RDW-SD, red cell distribution width-standard deviation; RDW-CV, red cell distribution width-corpuscular volume; MELD, Model for End-Stage Liver Disease; CTP, Child-Turcotte-Pugh; fL, femtoliters

Patient characteristics		Frequency	Percentage
Age group (years)	31-40	16	32
	41-50	19	38
	51-60	15	30
Gender	Male	43	86
	Female	7	14
RDW-SD (in fL)	<46	7	14
	46.1-50	8	16
	50.1-60	13	26
	60.1-70	13	26
	>70	9	18
RDW-CV (in percentage)	<14.6	7	14
	14.7-16.5	12	24
	16.6-18.5	12	24
	18.6-20.5	7	14
	>20.6	12	24
Ascites	Mild	7	14
	Moderate	39	78
	Severe	4	8
Encephalopathy grade	0	18	36
	1	5	10
	2	13	26
	3	10	20
	4	4	8
CTP score	5-6	6	12
	7-9	16	32
	10-15	28	56
MELD score	<9	8	16
	10-19	22	44
	20-29	11	22
	30-39	8	16
	>40	1	2

The correlation between ascites and encephalopathy grade with RDW-SD is presented in Table 2. Among the patients with mild ascites, three individuals had an RDW-SD value below 46 fL. In patients with moderate ascites, RDW-SD values of 50.1-60 and 60.1-70 fL were observed in 10 and 12 patients, respectively. Two patients with RDW-SD values of 50.1-60 fL exhibited severe ascites. Statistical analysis indicated a nonsignificant correlation between ascites and RDW-SD (p = 0.08). Among the 18 patients in encephalopathy grade 0, five patients each had RDW-SD values below 46 fL and in the range of 46.1-50 fL. In patients with encephalopathy grade 3, six individuals had an RDW-SD value exceeding 70 fL. However, no statistically significant correlation was observed between encephalopathy grade and RDW-SD (p = 0.056), as shown in Table 2.

**Table 2:** Ascites and encephalopathy grade with RDW-SD RDW-SD, red cell distribution width-standard deviation; fL, femtoliters

Variables		RDW-SD (fL)					Total	p-value
		<46	46.1-50	50.1-60	60.1-70	>70		
Ascites	Mild	3	3	1	0	0	7	0.08
	Moderate	4	5	10	12	8	39	
	Severe	0	0	2	1	1	4	
Encephalopathy grade	0	5	5	4	3	1	18	0.056
	1	1	1	2	1	0	5	
	2	1	1	5	4	2	13	
	3	0	0	1	3	6	10	
	4	0	1	1	2	0	4	

Table 3 presents the association between ascites and encephalopathy grade with RDW-CV. Three patients with mild ascites had an RDW-CV of less than 14.6%. Moderate ascites was observed in 10 patients with an RDW-CV ranging from 16.6% to 18.5% and in seven patients with an RDW-CV ranging from 18.6% to 20.5%. Among the patients with moderate ascites, two individuals with an RDW-CV of 16.6-18.5% exhibited severe ascites. A statistically significant correlation was found between ascites and RDW-CV values (p = 0.029). In the group of patients with encephalopathy grade 0, five patients had an RDW-CV of less than 14.6%, and seven patients had an RDW-CV ranging from 14.7% to 16.5%. Among the patients with encephalopathy grade 3, six individuals had an RDW-CV higher than 20.6%. However, no statistically significant correlation was observed between encephalopathy grade and RDW-CV values (p = 0.135), as shown in Table 3.

**Table 3:** Ascites and encephalopathy grade with RDW-CV RDW-CV, red cell distribution width-corpuscular volume

Variables		RDW-CV (percentage)					Total	p-value
		<14.6	14.7-16.5	16.6-18.5	18.6-20.5	>20.6		
Ascites	Mild	3	4	0	0	0	7	0.029
	Moderate	4	8	10	7	10	39	
	Severe	0	0	2	0	2	4	
Encephalopathy grade	0	5	7	3	1	2	18	0.135
	1	1	0	2	1	1	5	
	2	1	3	4	3	2	13	
	3	0	0	2	2	6	10	
	4	0	2	1	0	1	4	

A significant association was found between RDW-SD and CTP scores (p < 0.0001). Patients with lower CTP scores (5-6) exhibited lower RDW-SD values, whereas 19 patients with higher CTP scores had RDW-SD values exceeding 60.1 (Table 4). Furthermore, there was a statistically significant correlation between MELD scores and RDW-SD (p < 0.006). Among 22 patients with MELD scores ranging from 10 to 19, the majority (10 patients) had RDW-SD values in the range of 50.1-60 fL (Table 4). Only one patient had a MELD score exceeding 40, and in this patient, RDW-SD was observed to be above 70 fL (Table 4).

**Table 4:** CTP and MELD scores with RDW-SD CTP, Child-Turcotte-Pugh; MELD, Model for End-Stage Liver Disease; RDW-SD, red cell distribution width-standard deviation; fL, femtoliters

Variables		RDW-SD (fL)					Total	p-value
		<46	46.1-50	50.1-60	60.1-70	>70		
CTP score	5-6	4	2	0	0	0	6	<0.0001
	7-9	2	4	7	2	1	16	
	10-15	1	2	6	11	8	28	
MELD score	<9	4	4	0	0	0	8	0.006
	10-19	2	1	10	5	4	22	
	20-29	1	2	1	5	2	11	
	30-39	0	1	2	3	2	8	
	>40	0	0	0	0	1	1	

Table 5 illustrates the statistically significant correlation between RDW-CV values and CTP scores (p = 0.002). Among the 28 patients with higher CTP scores, 11 patients had RDW-CV values exceeding 20.6%. Conversely, patients with lower CTP scores (5-6) exhibited lower RDW-CV values. Additionally, a statistically significant association was observed between MELD scores and RDW-CV (p = 0.034), as shown in Table 5. Among the 22 patients with MELD scores ranging from 10 to 19, eight patients had RDW-CV values in the range of 16.6-18.5%. Only one patient had a MELD score exceeding 40, and in this patient, RDW-CV was observed to be above the range of 18.6-20.5% (Table 5).

**Table 5:** CTP and MELD scores with RDW-CV CTP, Child-Turcotte-Pugh; MELD, Model for End-Stage Liver Disease; RDW-CV, red cell distribution width-corpuscular volume

Variables		RDW-CV (percentage)					Total	p-value
		<14.6	14.7-16.5	16.6-18.5	18.6-20.5	>20.6		
CTP score	5-6	4	2	0	0	0	6	0.002
	7-9	1	5	6	3	1	16	
	10-15	2	5	6	4	11	28	
MELD score	<9	4	3	0	0	1	8	0.034
	10-19	2	5	8	4	3	22	
	20-29	1	2	1	2	5	11	
	30-39	0	2	3	0	3	8	
	41-50	0	0	0	1	0	1	

## Discussion

RDW, which measures the heterogeneity of RBC sizes, has been recognized as a valuable prognostic indicator in various cardiovascular conditions such as cardiac failure, coronary diseases, and acute myocardial infarction [14-16]. In a study by Lou et al., the correlation between RDW values and patients infected with hepatitis B was investigated, revealing a significant increase in RDW values among individuals with hepatitis B, with a positive association with disease severity [17]. In recent years, a notable disparity has emerged between the number of available liver donors and the growing population of liver disease patients awaiting transplantation. This presents a challenge in determining the optimal timing of surgery, as it can significantly impact both mortality and morbidity outcomes. Even a slight variation in the decision-making process could have adverse consequences for patients who would greatly benefit from transplantation [18,19].

RDW is a routinely measured parameter in complete blood counts (CBC) and reflects the variability in RBC size. While traditionally used as an indicator of anemia, emerging evidence suggests that RDW may also serve as a valuable marker for various systemic diseases, including liver disorders. This short note aims to explore potential mechanisms underlying the association between RDW and liver disorders. Liver disorders can impair hepatocellular function, leading to reduced synthesis and secretion of proteins involved in erythropoiesis, such as erythropoietin (EPO). EPO, produced mainly in the kidney and to a lesser extent in the liver, is essential for RBC production. Decreased EPO levels resulting from liver dysfunction may contribute to ineffective erythropoiesis, resulting in anisocytosis and increased RDW. In advanced liver disorders, such as cirrhosis, portal hypertension can develop due to increased resistance to blood flow within the liver. Portal hypertension can cause splenic congestion and enlargement, leading to hypersplenism and increased destruction of RBCs (hemolysis). Hemolysis contributes to the release of immature RBCs into circulation, resulting in a higher RDW.

The MELD score plays a crucial role in evaluating the mortality and morbidity risk and assessing the severity of cirrhosis in patients, aiding in the prioritization of candidates for transplantation. This score takes into account bilirubin levels, serum albumin levels, INR, and serum creatinine levels. It is calculated statistically with coefficients derived from these parameters on a continuous scale, without specific upper or lower limits [20,21]. On the other hand, the CTP score, developed over three decades ago, effectively assesses the short- and medium-term prognosis. In our study, we found a significant positive correlation between both the MELD score and the CTP score with RDW-SD and RDW-CV values. In recent years, numerous studies have highlighted the utility of RDW as a prognostic marker not only for liver diseases but also for various other conditions [22]. An elevated RDW can be attributed to impaired erythropoiesis or abnormal RBC survival, and this increase may be associated with factors such as oxidative stress, hypertension, malnutrition, or erythrocyte disorders.

In a retrospective study conducted by Zhu et al. on the Chinese population from 2014 to 2017, a significant correlation was observed between RDW and total bilirubin, albumin levels, and CTP scores in patients with HBV-related hepatocirrhosis [23]. The study found that RDW-SD and RDW-CV values decreased after antiviral therapy in HBeAg+ patients and those with chronic hepatitis B (CHB). Furthermore, the study concluded that RDW could serve as a valuable tool in distinguishing HBV-related hepatocirrhosis from CHB patients and inactive HBV carriers [23]. Similarly, in our study, we also identified a positive and significant correlation between RDW-SD and RDW-CV scores and prothrombin time, INR, CTP scores, and MELD scores. Additionally, there was a statistically significant negative correlation between serum albumin levels and both RDW-SD and RDW-CV scores.

Additional studies have provided evidence supporting a positive correlation between RDW and liver fibrosis and inflammation [22,23]. In a retrospective analysis of liver biopsy samples from 519 patients admitted to the Liver Disease Center at the First Affiliated Hospital of Fujian Medical University between January 2010 and December 2011, conducted by Xu et al., a gradual increase in RDW values with the progression of inflammation was observed. The study revealed that the risk of advanced inflammatory activity increased by 25.9% for every 1% rise in RDW values as a continuous variable in the univariate logistic regression analysis [24].

Limitations

The utilization of RDW for diagnostic and prognostic purposes in liver diseases is subject to certain limitations. RDW can be influenced by various factors such as age, sex, malnutrition, obesity, inflammation, and oxidative stress. In the study conducted by Xu et al., the relationship between HBV-related liver disease and RDW was explored [24]. The increase in RDW values can be attributed to three main reasons. Firstly, it can be attributed to the persistent inflammation in progressive liver fibrosis and the impact of pro-inflammatory cytokines on erythropoiesis, leading to the release of immature erythrocytes into circulation and subsequently raising RDW values [24]. Secondly, there is an observed increase in RDW among adolescents with higher BMI, potentially linked to the higher prevalence of metabolic syndrome in chronic hepatitis patients with cirrhosis compared to those without cirrhosis. Thirdly, endothelial dysfunction and inflammation associated with chronic kidney disorders can contribute to elevated RDW levels [24]. However, the precise mechanism underlying the correlation between increased RDW and the severity of liver disorders remains uncertain. 

The potential significance of RDW in enhancing our comprehension and management of CLDs and other chronic medical conditions should not be overlooked. Unlike other biomarkers, RDW assessment is a cost-effective option that eliminates the need for invasive procedures like biopsies [25]. Consequently, it is imperative to conduct further research to explore the potential of RDW as a dependable prognostic biomarker for emerging viruses. Additionally, efforts should be made to develop strategies that mitigate the limitations associated with utilizing this easily accessible predictive measure [25]. By gaining a deeper understanding of the precise pathophysiological changes contributing to elevated RDW levels, it becomes possible to identify confounding factors and potential disease pathways that could be targeted for modifying disease outcomes and improving the management of chronic medical conditions. Therefore, it is recommended that future studies employing randomized clinical trials be undertaken to ascertain the exact sensitivity and specificity of RDW as a prognostic and predictive marker in CLDs.

## Conclusions

The study findings suggest a positive correlation between increased RDW values and the severity of CLD in patients. Specifically, RDW values exhibit a direct relationship with both the MELD score and CTP score. Consequently, RDW holds promise as a potential and easily accessible tool for assessing the severity of CLD in patients.
